# The Serum Lipid Profile of Relapsing Multiple Sclerosis Differs Reproducibly From Healthy Controls

**DOI:** 10.1111/jnc.70285

**Published:** 2025-11-05

**Authors:** Lisa Shi, Laura Ghezzi, Georgia Watt, Drishya Mainali, Dana Perantie, Chiara Fenoglio, Collin Tran, Alexander Dupuy, Freda Passam, Monokesh K. Sen, Humphrey Chan, Samuel Kwok, Chenyu Wang, Michael Barnett, Todd Hardy, Laura Piccio, Anthony S. Don

**Affiliations:** ^1^ Charles Perkins Centre, the University of Sydney Sydney New South Wales Australia; ^2^ Brain and Mind Centre, the University of Sydney Sydney New South Wales Australia; ^3^ School of Medical Sciences, Faculty of Medicine and Health The University of Sydney Sydney New South Wales Australia; ^4^ Department of Biomedical, Surgical and Dental Sciences University of Milan Milan Italy; ^5^ Fondazione IRCCS Ca' Granda Ospedale Maggiore Policlinico Milano Italy; ^6^ Department of Neurology, Washington University, School of Medicine St Louis Missouri USA; ^7^ Royal Prince Alfred Hospital Sydney New South Wales Australia; ^8^ Central Clinical School, Faculty of Medicine and Health The University of Sydney Sydney New South Wales Australia; ^9^ Department of Haematology Royal Prince Alfred Hospital Sydney New South Wales Australia; ^10^ Illawarra Shoalhaven Local Health District New South Wales Australia; ^11^ Napean‐Blue Mountains Local Health District New South Wales Australia; ^12^ Sydney Neuroimaging Analysis Centre Sydney New South Wales Australia; ^13^ Department of Neurology Concord Hospital Sydney New South Wales Australia

**Keywords:** biomarker, lipid, lipidomics, lysophospholipid, multiple sclerosis, sphingolipid

## Abstract

Blood biomarkers that correlate with inflammatory demyelination or neurodegeneration are needed for early diagnosis and disease monitoring in multiple sclerosis (MS). This study aimed to identify serum lipids that are reproducibly altered in relapsing–remitting MS (RRMS) and correlated with indicators of disease severity. Lipids were quantified using liquid chromatography–tandem mass spectrometry in a discovery cohort of 47 RRMS, 42 other neurological disorders, and 30 healthy control serum samples, and a validation cohort of 81 RRMS, 18 progressive MS and 33 control samples. Levels were compared between groups using ANOVA adjusted for age, sex, collection site, and treatment status. Correlations of lipids with age, sex, relapse status, and expanded disability severity scale (EDSS) and multiple sclerosis severity scale (MSSS) scores were determined. Eight lysophosphatidylcholines (LPC), five lysophosphatidylethanolamines (LPE), two acylcarnitines, five diacylglycerols, three sphingomyelins (SM), one ceramide, and one hexosylceramide were significantly higher in both RRMS cohorts compared to healthy controls. Random forest models including five lipids predicted RRMS with 83% sensitivity and 77% specificity (discovery), or 89% sensitivity and 61% specificity (validation cohort). Three acylcarnitines, one brain‐enriched ceramide (d18:1/18:0), and two myelin‐enriched hexosylceramides were positively correlated with EDSS scores in the validation cohort, of which two were also correlated with MSSS scores. However, these associations were not observed in the discovery cohort. The neuron‐enriched lipid SM(d18:1/18:0) was significantly higher during relapse in the validation and combined cohorts. In conclusion, this study identifies a set of 25 lipids that are reproducibly elevated in serum of people with RRMS. Higher serum LPCs, LPEs, diacylglycerols and acylcarnitines may reflect a peripheral lipolysis signature, while higher levels of neuron‐enriched sphingolipids could reflect neurodegeneration. Future studies should investigate if these serum lipids predict brain atrophy and disability progression.

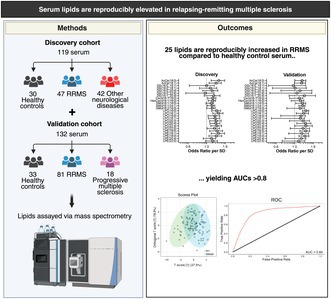

## Graphical abstract

Blood biomarkers that correlate with inflammatory demyelination or neurodegeneration are needed for early diagnosis and disease monitoring in multiple sclerosis. This study employed lipidomic analysis to identify serum lipids that are reproducibly altered in relapsing–remitting multiple sclerosis (RRMS) across two independent sample cohorts, finding that 25 lipids are reproducibly increased in the serum of RRMS relative to healthy control subjects. Higher serum lysophosphatidylcholines, lysophosphatidylethanolamines, diacylglycerols and acylcarnitines may reflect a peripheral lipolysis signature, while higher levels of neuron‐enriched sphingolipids could reflect neurodegeneration. Further studies are needed to determine if levels of these lipids predict brain atrophy and disability progression.

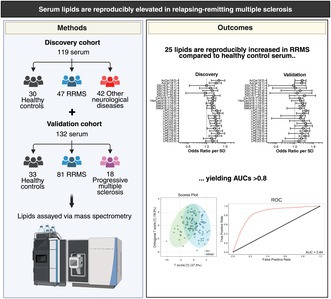

**Abbreviations**
AcCa: acylcarnitine
ADEM: acute disseminated encephalomyelitis
AI
: artificial intelligence
BHT
: butylated hydroxytoluene
C1P: ceramide 1‐phosphate
Cer: ceramide
CNS: central nervous system
CSF: cerebrospinal fluid
DG: diacylglycerol
EDSS
: Expanded Disability Status Scale
FDR: false discovery rate
GalCer: galactosylceramide
GFAP: glial fibrillary acidic protein
GluCer: glucosylceramide
HC: healthy control
Hex2Cer: dihexosylceramide
HexCer: hexosylceramide
LPC: lysophosphatidylcholine
LPE: Lysophosphatidylethanolamine
MOGAD: myelin oligodendrocyte glycoprotein antibody disease
MRI: magnetic resonance imaging
MS: multiple sclerosis
MSSS: multiple sclerosis severity score
NfL: neurofilament light
NMOSD
: neuromyelitis optica spectrum disorder
OND: other neuroinflammatory disease
oPLS‐DA: orthogonal partial least squares discriminant analysis
OR: odds ratio
PC
: phosphatidylcholine
PE
: phosphatidylethanolamine
PEp
: phosphatidylethanolamine plasmalogen
PLA_2_

: phospholipase A_2_

PMS: progressive multiple sclerosis
PS: phosphatidylserine
ROC: receiver operator curve
RRMS
: relapsing–remitting multiple sclerosis
SD: standard deviation
SHexCer
: sulfatide
SM
: sphingomyelin
VIP: Variable Importance in Projection

## 1 Introduction

MS diagnosis relies on clinical presentation and the presence of central nervous system (CNS) white matter lesions on magnetic resonance imaging (MRI) that are disseminated in time and space (Brownlee et al. 2017). IgG oligoclonal bands in cerebrospinal fluid (CSF) are currently the only fluid biomarker that supports MS diagnosis, and are associated with conversion from clinically isolated syndrome to MS, intrathecal inflammation, cortical lesion load and greater brain atrophy (Jin et al. 2023). Oligoclonal bands are absent in 10% of MS cases and lack specificity, as they are also observed in 20%–30% of neuromyelitis optica spectrum disorder (NMOSD) and myelin oligodendrocyte glycoprotein antibody disease (MOGAD) cases, and encephalitis (Jin et al. 2023). Relapses and disease progression are monitored by assessing clinical signs, symptoms, and new lesions on MRI. However, MRI measures detect neurological damage that has already occurred and do not necessarily correlate with clinical severity (Hemond and Bakshi 2018).

Blood biomarkers that predict disease progression and treatment response might detect changes in disease activity prior to MRI and would allow monitoring of treatment efficacy over time. Numerous candidates are emerging, such as neurofilament light chain (NfL), a cytoskeletal component of axons released upon neuronal damage (Thebault et al. 2021); and glial fibrillary acidic protein (GFAP), a marker of astrocyte activation (Högel et al. 2020). Both of these are generalised biomarkers of neurodegeneration that are not specific to MS (also increased in other neurodegenerative diseases) and their levels are affected by age and body mass index (BMI) (Benkert et al. 2022; Högel et al. 2020; Manouchehrinia et al. 2020), making it difficult to establish thresholds. Higher serum NfL predicts clinical onset of MS, higher relapse rates, more severe relapses, greater cortical atrophy and MRI lesion activity, poorer cognitive function and faster disease progression (Thebault et al. 2021). Serum GFAP levels are associated with worse Expanded Disability Status Scale (EDSS) scores, reduced cortical grey matter volume and higher lesion load (Högel et al. 2020). While some studies report elevated GFAP levels in MS patients, others find no significant differences (Abdelhak et al. 2022).

Myelin, the primary target of autoimmune attack and degeneration in MS, is 70%–80% lipid (dry mass) and highly enriched for certain lipids such as galactosylceramide (GalCer) and sulfatide (SHexCer) (Quarles et al. 2006). Since GalCer accounts for 20% of myelin lipids (Quarles et al. 2006), it is highly abundant in the CNS. However, the structural isomer of GalCer, glucosylceramide (GluCer), is much more abundant in blood plasma (Marian et al. 2025). As they cannot easily be distinguished with mass spectrometry, GalCer and GluCer are collectively referred to as hexosylceramide (HexCer). Higher serum and plasma HexCer levels have been reported in people with progressive MS, and were correlated with retinal nerve fibre thinning (Filippatou et al. 2021) and higher rates of brain atrophy (Amatruda et al. 2020). Serum ceramide (Cer) levels are also higher in MS (Filippatou et al. 2021; Villoslada et al. 2017), and particular ceramides have been associated with higher EDSS scores and may predict functional decline (Filippatou et al. 2021; Virupakshaiah et al. 2023). In contrast, reduced levels of the glycerophospholipids phosphatidylcholine (PC) and phosphatidylethanolamine (PE) have been reported in the plasma (Lattau et al. 2024) and serum (Ferreira et al. 2021; Villoslada et al. 2017) of people with MS. Further lipidomic studies are needed to reconcile inconsistencies in the literature and identify lipids that are reproducible and reliable biomarkers of MS disease activity. This study aimed to characterise the serum lipidomic profile of RRMS relative to healthy controls (HC) in two distinct sample cohorts, and determine if any serum lipids are reproducibly associated with relapses and disease severity scores.

## 2 Methods

### 2.1 Serum Samples

This study was not pre‐registered, and was approved by the University of Sydney Human Ethics Committee (2021_295 and 2021_059) and the Washington University Institutional Review Board (201104379). Discovery cohort samples were from the Washington University Multiple Sclerosis and Nervous System Diseases Repository and Database (Table S1A). Validation cohort samples were from the IRCCS Ospedale Maggiore Policlinico, Milan (Milan Area 2 Ethics Committee, parere 1068_2021bis) and The University of Sydney (Table S1B). Informed consent was obtained from all participants. Samples were collected between 2005 and 2023, aliquoted, and stored at −80°C. Only samples with one or two freeze–thaw cycles were used. Diagnosis of MS was according to the 2017 McDonald criteria (Thompson et al. 2018). All MS sample donors in the discovery cohort were untreated at time of sample collection. In the validation cohort, 48 MS sample donors were untreated and 51 were treated at the time of collection. EDSS scores were available for 39 of 48 MS cases in the discovery cohort, and 97 of 99 cases in the validation cohort. Multiple Sclerosis Severity Scale (MSSS) scores were calculated from EDSS scores and disease duration (Roxburgh et al. 2005). Based on the discovery cohort, a sample size of 102 MS cases for the validation cohort was estimated to provide 95% power to identify correlation coefficients of 0.45 at *p* < 0.001. In reporting this study, we have followed the Strengthening the Reporting Observational studies in Epidemiology—Molecular Epidemiology (STROBE‐ME) guidelines.

### 2.2 Lipid Extraction

Lipids were extracted from 20 μL serum samples by adding 650 μL of 1‐butanol, and 650 μL of methanol containing internal standards (Table 1) and 0.01% (w/v) butylated hydroxytoluene (BHT) (Shi et al. 2024). Samples were vortexed, sonicated for 2 h at 4°C in a sonicating water bath, and centrifuged at 16 000 *g* for 10 min. The supernatants were collected in glass vials and the pellets were re‐extracted using 1:1 (v/v) 1‐butanol/methanol with 1 h sonication. The supernatants were combined and dried overnight in a Savant SpeedVac SC210 at 35°C, after which the extracted lipids were reconstituted in 200 μL 80% methanol/20% HPLC‐grade water with 0.1% HCOOH, 10 mM NH4^+^HCOO^−^ and 0.01% BHT. These were centrifuged at 2000 *g* for 10 min to pellet debris, transferred to glass vials and stored at −80°C.

**TABLE 1:** Internal standards used for lipid extractions.

Standard	Supplier	Cat. #	*n* moles per sample	Precursor ion (*m/z*)	Product ion (*m/z*)	Retention time (min)
PC(19:0/19:0)	Avanti polar lipids	850367P	5	818.7 [M+H]^+^	184.1	13.3
SM(d18:1/17:0)	Cayman chemical	25592	2	717.6 [M+H]^+^	184.1	11.3
GluCer(d18:1/12:0)	Avanti polar lipids	860543	2 (discovery cohort)	644.6 [M+H]^+^	264.3	9.7
GluCer(d18:1/17:0)	Avanti polar lipids	860569P	2 (validation cohort)	714.6 [M+H]^+^	264.3	11.5
PS(17:0/17:0)	Avanti polar lipids	840028	2	762.5 [M−H]^−^	269.2	11.5
PE(17:0/17:0)	Avanti polar lipids	830756	2	718.5 [M−H]^−^ 720.6 [M−H]^+^	269.2 [−H] 579.5 [+H]	12.3
PG(17:0/17:0)	Avanti polar lipids	830456	2 (discovery cohort)	749.5 [M−H]^−^	269.2	11.5
CE(17:0)	Avanti polar lipids	700186	2 (discovery) 5 (validation)	656.6 [M+NH_4_]^+^	369.4	15.8
TG(17:0/17:0/17:0)	Cayman chemical	19722	2 (discovery cohort)	866.8 [M+NH_4_]^+^	579.5	15.7
TG(16:0/16:0/16:0)‐d31	Cayman chemical	23334	5 (validation cohort)	918.4 [M+NH_4_]^+^	612.8	15.4
PA(17:0/17:0)	Avanti polar lipids	830856	1	675.5 [M−H]^−^	269.2	12.4
PI(18:1/15:0)‐d7	Avanti polar lipids	791641C	1	828.6 [M−H]^−^	241.0	10.4
Cholesterol‐d7	Avanti polar lipids	791645	1 (discovery) 5 (validation)	376.4 [M+H‐H_2_O]^+^	161.1	9.4
Cer(d18:1/17:0)	Avanti polar lipids	860517P	0.5	552.5 [M+H]^+^	264.3	12
LacCer(d18:1/12:0)	Avanti polar lipids	860545P	0.5	806.6 [M+H]^+^	264.3	9.5
SHexCer(d18:1/17:0)	Avanti polar lipids	860572P	0.5	794.6 [M+H]^+^	264.3	10.3
C1P(d18:1/12:0)	Avanti polar lipids	860531	0.5	562.4 [M+H]^+^	264.3	9.3
LPC(17:0)	Avanti polar lipids	855676	0.5	510.4 [M+H]^+^	184.1	6
LPE(17:1)	Avanti polar lipids	856707	0.5	466.2 [M+H]^+^	325.2	4.1
DG(18:1/15:0)‐d7	Avanti polar lipids	791647	0.5	605.6 [M+NH_4_]^+^	299.3	12.4
LPS(17:1)	Avanti polar lipids	858141	0.5 (discovery cohort)	508.3 [M−H]^−^	421.2	2.8
Sph(d17:1)	Avanti polar lipids	860640P	0.2	286.3 [M+H]^+^	250.2	2.9
S1P(d17:1)	Cayman chemical	22498	0.2	366.2 [M+H]^+^	250.2	2.4
AcCa(16:0)‐d3	Avanti polar lipids	55107	0.2	403.4 [M+H]^+^	85.0	5.2
GM3(d18:1/18:0)‐d5	Avanti polar lipids	860073	0.2 (discovery cohort)	1185.8 [M−H]^−^	290.1	10.6

### 2.3 Lipid Quantification

Lipids were detected by multiple reaction monitoring on a TSQ Altis triple quadrupole mass spectrometer with a Vanquish HPLC system and a 2.1 × 100 mm Waters Acquity CSH C18 column (Shi et al. 2024). The flow rate was 0.28 mL/min with a binary solvent system comprising mobile phase A: 60% acetonitrile/40% water with 10 mM ammonium formate and 0.1% formic acid; and B: 10% acetonitrile/90% isopropanol with 10 mM ammonium formate and 0.1% formic acid. Run time was 25 min: 0–3 min, 20% B; 3–5.5 min, ramp to 45% B; 5.5–8 min, ramp to 65% B; 8–13 min ramp to 85% B; 13–14 min, ramp to 100% B; 14–20 min, hold at 100% B; 20–20.5 min, decrease to 20% B; 20.5–25 min hold at 20% B. Samples were randomised, with each run in positive and negative ion mode back‐to‐back. Every fifth injection was a solvent blank. Peaks were integrated using Tracefinder 5.1 (ThermoFisher Scientific). Only peaks present in at least 70% of the samples were quantified. All quantified peaks had a minimum of 10 consecutive scans, with an area greater than 1000 units. Lipid identifications were based on precursor and product ion pairs (Table S2), retention time vs. *m/z* plots (Hejazi et al. 2011), and high‐resolution untargeted lipidomic data collected on a ThermoFisher Q‐Exactive HF‐X mass spectrometer using the same HPLC, column and solvents. The concentration of each lipid (nM) was estimated by normalising to its class‐specific internal standard (Table 1), multiplying by the amount of internal standard added, and dividing by the sample volume.

### 2.4 Phospholipase A_2_ (PLA_2_ ) Activity

A spectrophotometric assay (Petrovic et al. 2001) was used to measure PLA_2_ activity in serum samples from the discovery (*n* = 117) and validation (*n* = 132) cohorts. Briefly, the chromogenic substrate 4‐nitro‐3‐octanoyloxy‐benzoic acid was reconstituted at 4 mg/mL in 150 mM KCl, 10 mM CaCl_2_, 50 mM Tris–HCl, pH 7.5, sonicated in a water bath for 10 s, vortexed, centrifuged (2000 *g*, 2 min) at RT, and the supernatant was collected as substrate solution. Serum samples (5 μL) were diluted 1:20 into substrate solution (95 μL) and incubated for 1 h at RT. Absorbances at 425 and 600 nm were read on a HT‐FRET TECAN Infinite M1000 PRO microplate reader. PLA_2_ activity was calculated as follows: PLA_2_ activity [μmol/h/mL] = [30 min (A425_nm_–A600_nm_)—0 min (A425_nm_–A600_nm_)] × 2 × 0.07862 × (1/serum volume). The correction factor (0.07862) represents the concentration of product producing an absorbance of 1.0 at 425 nm.

### 2.5 AI‐Assisted MRI Metrics

Artificial intelligence (AI)‐supported MRI measures were derived for a subset of the validation cohort MS samples from Sydney, including only samples for which an MRI had been performed within one year of blood draw (*n* = 40). A clinically validated commercial tool, iQ‐Solutions, was used to analyse brain MRI scans in Digital Imaging and Communications in Medicine format, producing measurements of T2 lesion number, total lesion volume, normalised whole brain volume, normalised thalamic volume, normalised grey matter volume and annualised percentage brain volume change (available for 35 cases) (Barnett et al. 2023).

### 2.6 Statistical Analysis

R studio (version 2024.04.1 + 748) was used for statistical analyses. Lipids not detected in particular samples were assigned a value of 1/5 of the minimum concentration for that species, provided they were absent in no more than 30% of the samples. Lipid concentrations and clinical covariates were log_10_‐transformed to improve normality, which was assessed with the Anderson‐Darling test and histograms. Lipid levels were compared across disease groups using one‐way ANOVA adjusted for age, sex, collection site and treatment (the latter two only in the validation cohort), followed by Tukey's post hoc test. Odds ratios were derived from logistic regression comparing lipid levels between RRMS and HC, adjusted for age, sex, collection site and treatment. Odds ratios per standard deviation (SD) were calculated using the odds ratio to the power of the population SD for that lipid. Correlations to clinical and AI‐supported MRI parameters were tested using Pearson's or Spearman's analyses. Partial correlations were used to account for the effects of age and collection site in the discovery and validation cohorts, respectively, on associations between lipids and EDSS/MSSS. Outliers determined by Grubb's test were excluded. *p* values arising from ANOVA or correlation analyses were corrected for false discovery rate (FDR) using the Benjamini–Hochberg method, with *q* < 0.05 considered significant.

Random forest models were performed with the *ClassifyR*, *dplyr*, *devtools*, *S4Vectors* and *ranger* packages in R, generating selected features, receiver operator curves (ROCs), balanced accuracies and confusion matrices. MetaboAnalyst 5.0 was used for orthogonal partial‐least squares discriminant analyses (oPLS‐DA) and to generate Variable Importance in Projection (VIP) plots. Bar and scatter plots were created using GraphPad Prism (version 10). Heatmaps were created in MetaboAnalyst 5.0, using the Euclidean distance measure with Ward clustering by group.

## 3 Results

### 3.1 Cohort Characteristics

To identify serum lipids whose levels differ in RRMS compared to HC and other neurological disorders (OND), levels of 208 lipids (Table S3) were first analysed in a set of 30 HC, 47 RRMS, and 42 OND serum samples from the Multiple Sclerosis and Nervous System Diseases Repository and Database at Washington University in St Louis (Table 2). A validation cohort comprising 33 HC, 81 RRMS, and 18 progressive MS (PMS) samples (Table 3) from the Universities of Milan and Sydney was then analysed to confirm associations of lipids with RRMS and EDSS/MSSS scores, as well as age and sex. Since the lipids showing significant associations in the discovery cohort were all detected with positive ion mode mass spectrometry, only positive ions (156 lipids) were analysed in the validation cohort. No significant differences in age and sex were observed between disease groups in the discovery cohort. In the validation cohort, PMS cases were on average 17 and 19 years older than HC (*p* < 0.0001) and RRMS (*p* = 0.0005), respectively (one‐way ANOVA: *F* (3, 113) = 15, *p* < 0.0001).

**TABLE 2:** Discovery cohort demographics and clinical characteristics.

	Study population	HC	OND	RRMS
Sample size, *n*	119	30	42^a^	47^b^
Age (years), mean ± SD	41 ± 14	38 ± 14	43 ± 15	41 ± 14
Female, *n* (%)	76 (64%)	15 (50%)	29 (71%)	32 (67%)
Median EDSS (range)	—	—	—	2 (1–6)
BMI (kg/m^2^), mean ± SD	29 ± 7	ND	30 ± 7	29 ± 8

*Note:* All participants were untreated, except for three with NMOSD who were on systemic corticosteroids at the time of sampling.

Abbreviation: ND, not determined.

^a^ OND cases (*n*): *NMOSD (10), headache/migraine (5), *MOGAD (3), *neurosarcoidosis (3), *transverse myelitis (3), *meningitis (2), *acute disseminated encephalomyelitis (2), *rheumatoid arthritis (1), *vasculitis (1), *chronic lymphocytic inflammation with pontine perivascular enhancement responsive to steroids (1), *chronic inflammatory demyelinating polyneuropathy (1), subjective memory complaints (1), syringomyelia (1), tectal glioma (1), progressive supranuclear palsy (1), pseudotumor cerebri (1), *CNS lymphoma (1), small fibre neuropathy (1), *mononeuritis multiplex (1), neuropathy (1), *acute motor axonal neuropathy (1). *Indicates a definitively inflammatory or immune‐mediated disease.

^b^ 10 RRMS cases were in relapse at the time of sampling, and 37 were in remission.

**TABLE 3:** Validation cohort demographics and clinical characteristics.

	Study population	HC	RRMS	PMS
Sample size, *n*	132	33	81^b^	18
Age (years), mean ± SD	44 ± 15	43 ± 13	41 ± 14	50 ± 15^a^
Female, *n* (%)	76 (64%)	21 (64%)	55 (68%)	8 (44%)
Collection in Sydney/Milan	92/40	33/0	44/37	15/3
Disease duration (years), mean (SD)	—	—	9 ± 9	13 ± 15
EDSS score, median (range)	—	—	2 (0–7)	5 (2–8.5)
BMI (kg/m^2^), mean ± SD	25 ± 5	24 ± 4	26 ± 5	26 ± 6
Treated, *n* (%)	—	—	42 (52%)	9 (50%)
Disease modifying therapeutic (*n*)	—	—	Natalizumab (12), dimethyl fumarate (11), ocrelizumab (10), fingolimod (6), alemtuzumab (1), glatiramer acetate (1), steroid (1)	Ocrelizumab (4), siponimod (4), natalizumab (1)

^a^ Significantly higher in PMS compared to RRMS and HC (*F* (3, 113) = 15.4, *p* < 0.0001).

^b^ Of 81 RRMS cases, 13 were in relapse at the time of sampling.

### 3.2 Association of Serum Lipids With Age, Sex, Treatment, and Collection Site

In multivariate models assessing the effect of disease group, age, sex, collection site and treatment on lipid levels, 39 lipids were significantly associated (*q <* 0.05) with age in the discovery cohort, and two lipids in the validation cohort (Tables S3 and S4). However, there were no commonalities. Six lipids were significantly higher in females compared to males in the discovery cohort, of which SM(d18:2/20:0) and SM(d18:2/24:1) were also significantly higher in the validation cohort. No other lipids differed by sex in the validation cohort. No lipids were associated with BMI in the discovery cohort (*n* = 88), whereas six SMs, two ceramides, and one acylcarnitine (AcCa) were positively correlated with BMI in the validation cohort (*n* = 90, Table S5).

Collection site for the validation cohort (Sydney or Milan) had a significant effect on the levels of 15 lipids, and was therefore included as a covariate (Table S3). In *t*‐tests comparing MS cases in the validation cohort that were treated with disease‐modifying therapies at the time of collection (*n* = 51) to those that were not (*n* = 48), two dihexosylceramides Hex2Cer(d18:1/16:0) (*t* = 3.7, *q* = 0.029) and Hex2Cer(d18:1/18:0) (*t* = 3.3, *q* = 0.048), ceramide 1‐phosphate C1P(d18:1/24:1) (*t* = 4.2, *q* = 0.0081), and AcCa(18:1) (*t* = 3.4, *q* = 0.048) were reduced with treatment (*q* < 0.05). However, this effect was lost in the multivariate regression model.

### 3.3 Twenty‐Five Lipids Were Reproducibly Higher in RRMS

**FIGURE 1:**
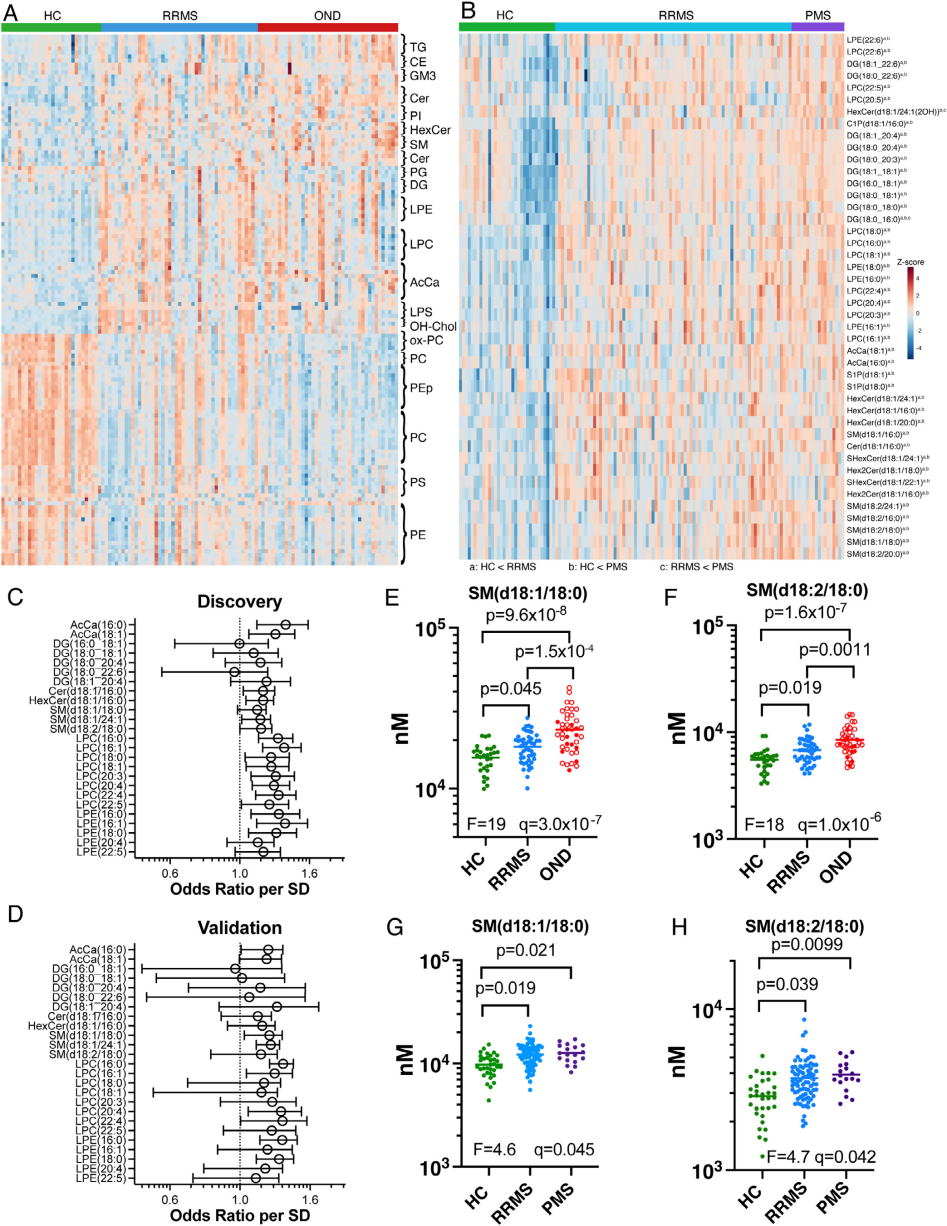
Twenty‐five lipids are reproducibly elevated in RRMS compared to HC serum. (A) Heatmap of lipids significant (*q* < 0.05) in one‐way ANOVA adjusted for age and sex in the discovery cohort. HC, healthy controls; OND, other neurological disorder; RRMS, relapsing–remitting multiple sclerosis. (B) Heatmap of lipids significant (*q* < 0.05) in one‐way ANOVA adjusted for age, sex, collection site and treatment in the validation cohort. PMS, progressive MS. Superscripts (a, b, c) indicate significance in Tukey's post‐tests at *p* < 0.05, a: HC < RRMS, b: HC < PMS, c: RRMS < PMS. (C, D) Forest plots showing odds ratios per SD, with 95% confidence intervals, for the 25 lipids that were reproducibly increased in RRMS compared to healthy controls (HC) in both the (C) discovery and (D) validation cohorts (as determined by Tukey's post‐test following one‐way ANOVA shown in A and B). (E–H) Levels of SM(d18:1/18:0) (E, G) and SM(d18:2/18:0) (F, H) in the discovery (E, F) and validation (G, H) cohort samples. ANOVA *F* and *q* values are shown on the graphs, as well as results of Tukey's post‐test. In the OND group, neuroinflammatory diseases are indicated by open circles. AcCa, acylcarnitine; CE, cholesterol ester; Cer, ceramide; DG, diacylglycerol; GM3, ganglioside GM3; HexCer, hexosylceramide; Hex2Cer, dihexosylceramide; LPC, lysophosphatidylcholine; LPE, lysophosphatidylethanolamine; LPS, lysophosphatidylserine; PC, phosphatidylcholine; PE, phosphatidylethanolamine; PEp, phosphatidylethanolamine plasmalogen; PG, phosphatidylglycerol; PGPC, 1‐palmitoyl‐2‐glutaryl‐sn‐glycero‐3‐phosphocholine; PI, phosphatidylinositol; POVPC, 1‐O‐palmitoyl‐2‐O‐(5‐oxovaleryl)‐sn‐glycero‐3‐phosphocholine; PS, phosphatidylserine; SM, sphingomyelin; Sph, sphingosine; SHexCer, sulfatide; S1P, sphingosine‐1‐phosphate; TG, triacylglycerol.

In the discovery cohort, 133 lipids differed significantly in abundance (*q* < 0.05) between the HC, RRMS and OND groups (Figure 1A, Table S3). Of these, 119 differed significantly between HC and OND, 109 between HC and RRMS, and 45 between RRMS and OND. In general, glycerophospholipids were lower in RRMS and OND compared to HC, whereas sphingolipids, lysophospholipids, neutral glycerolipids and AcCa's were higher. Eight sphingolipids and three TG species were significantly higher in the OND compared to both the RRMS and HC groups, while 30 glycerophospholipid and one LPS species were significantly lower in OND compared to both RRMS and HC. Notably, all 11 PE plasmalogens (PEp) measured were significantly lower in OND compared to the other two groups, whereas both sphingosine and dihydrosphingosine were higher.

In the validation cohort, 44 lipids were significantly different (*q* < 0.05) between HC, RRMS and PMS (Figure 1B, Table S4). All 44 lipids, encompassing sphingolipids, lysophospholipids, AcCa's and diacylglycerols (DGs), were significantly higher in PMS compared to HC, and 43 were higher in RRMS compared to HC, while DG(18:0_16:0) and HexCer(d18:1/24:1(2OH)) were higher in PMS compared to RRMS.

**FIGURE 2:**
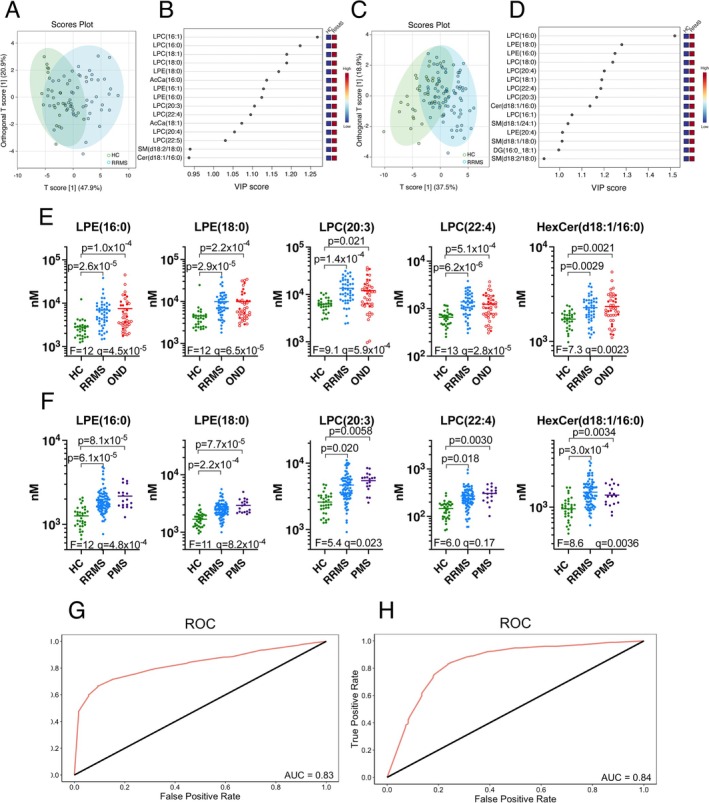
Higher serum LPC and LPE levels differentiate RRMS from HC. (A–D) oPLS‐DA score plots (A, C) and variable importance in the projection (VIP) plots (B, D) for differentiation of HC (green) from RRMS (blue) samples in the discovery (A, B) and validation (C, D) cohorts, based on the 25 lipids that were reproducibly higher in RRMS lipid profiles. (E, F) Levels of the five lipid species that best differentiated RRMS and HC samples, based on random forest models, in the discovery (E) and validation (F) cohorts. ANOVA *F* and *q* values, and significant differences arising from Tukey's post‐test are shown. Line represents mean for each group. In the OND group, neuroinflammatory diseases are indicated by open circles. (G, H) Receiver operating characteristic curves showing diagnosis of RRMS relative to HC in the discovery (G) and validation (H) cohorts. Curves were derived from random forest models using the five lipids shown in (E) and (F).

Comparing RRMS to HC across both cohorts, 25 lipids comprising two AcCa, five DG, one Cer, one HexCer, three SM, eight LPC and five LPE species were reproducibly higher in RRMS at *q* < 0.05 (Figure 1C,D). No lipids were reproducibly lower. Of these 25 lipids, SM(d18:1/18:0) and SM(d18:2/18:0) were significantly higher in OND compared to RRMS (Figure 1E,F), and none differed significantly between RRMS and PMS (Figure 1G,H). The highest SM(d18:1/18:0) levels were attributed to seven NMOSD, one neurosarcoidosis, and two acute disseminated encephalomyelitis (ADEM) samples (OND group), and levels were significantly higher in NMOSD compared to RRMS samples in adjusted ANOVA models (*F* (4, 112) = 13, *p* = 7.9 × 10^−9^, *q* = 4.7 × 10^−8^). Using the set of 25 lipids that were reproducibly higher in RRMS, oPLS‐DA models produced a partial separation between HC and RRMS in both cohorts, with LPC and LPE species identified as the most important features (Figure 2A–D). In random forest models using these 25 lipids, LPE(16:0), LPC(20:3), LPC(22:4), LPE(18:0) and HexCer(d18:1/16:0) contributed substantially to diagnostic accuracy in both cohorts (Figure 2E,F). A combination of these five lipids yielded a diagnostic accuracy of AUC = 0.83 (83% sensitivity and 77% specificity) for the discovery cohort, and AUC = 0.84 (89% sensitivity and 61% specificity) for the validation cohort (Figure 2G,H, Table S6).

**FIGURE 3:**
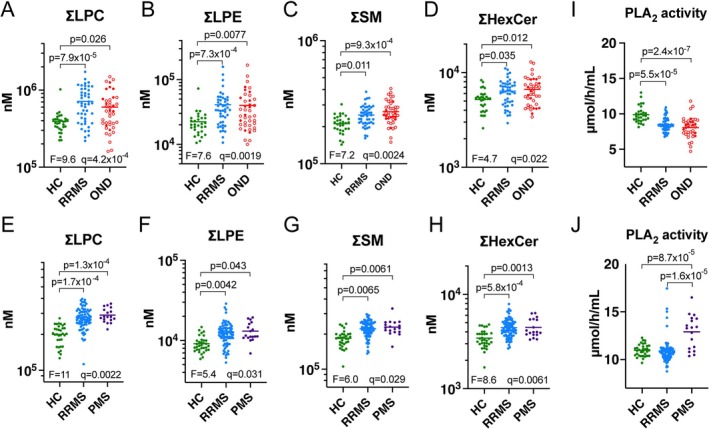
Total serum LPC, LPE, SM, and HexCer are reproducibly higher in RRMS. Total levels for lipid classes that were significantly higher in RRMS compared to HC in both discovery (A–D) and validation (E–H) cohorts. (I, J) PLA_2_ activity in discovery (I) and validation (J) cohort samples. Two OND cases from the discovery cohort were not included due to insufficient serum for this assay. ANOVA *F* statistics, *p* values and significant differences arising from Tukey's multiple comparisons are shown (*p* < 0.05). Line represents mean. In the OND group, known neuroinflammatory diseases are indicated by open circles.

When analysed as lipid class totals (sum of lipid species within each class), total LPC, LPE, SM, and HexCer were higher in RRMS compared to HC across both cohorts (Figure 3A–H and Tables S7 and S8).

### 3.4 PLA_2_ Activity is Not Increased in RRMS

We next tested if increased serum LPC and LPE levels in RRMS could be attributed to PLA_2_ activity, which hydrolyses the sn‐2 ester bond of PC/PE, releasing LPC/LPE and a fatty acid (Petrovic et al. 2001; Siroos et al. 2013). The mean PLA_2_ activity in serum from HC (*n* = 63) was 11.9 ± 1.1 μmol/h/mL, consistent with previous findings using the same assay (Petrovic et al. 2001). Contrasting with the increased LPC and LPE levels in the RRMS and OND groups, PLA_2_ activity was significantly lower in RRMS and OND compared to the HC group in the discovery cohort (one‐way ANOVA, *F* (2, 114) = 19.8, *p* < 0.0001) (Figure 3I). Mean PLA_2_ activity was also lower in the RRMS compared to HC samples in the validation cohort (not significant), but higher in PMS compared to RRMS and HC (Kruskal‐Wallis test, H(2) = 20.2, *p* < 0.0001) (Figure 3J). PLA_2_ activity did not correlate with lysophospholipid levels in either cohort.

### 3.5 Lysophospholipid, Sphingolipid and Triglyceride (TG) Levels are Correlated Between Serum and CSF

Correlations between CSF and serum levels of 144 lipid species were assessed in 60 samples from the discovery cohort for which CSF lipid levels were recently reported (Shi et al. 2024), resulting in 19 significantly correlated lipids (*q* < 0.05) (Table 4). Lysophospholipids were inversely correlated, whereas sphingolipids and TGs were positively correlated. Due to the limited number of matched CSF and serum samples in the validation cohort (*n* = 29), no lipids passed the FDR threshold.

**TABLE 4:** Lipid correlations between CSF and serum.

Lipid	Discovery			Validation		
	*r*	*p*	*q*	*r*	*p*	*q*
LPS(16:0)	−0.58	5.2 × 10^−7^	7.5 × 10^−5^			
LPC(18:1)	−0.49	3.9 × 10^−5^	1.9 × 10^−3^	0.16	4.2 × 10^−1^	7.7 × 10^−1^
LPE(18:1)	−0.49	4.1 × 10^−5^	2.0 × 10^−3^	0.01	9.5 × 10^−1^	9.7 × 10^−1^
LPC(18:2)	−0.47	9.6 × 10^−5^	3.5 × 10^−3^	0.35	6.0 × 10^−2^	6.7 × 10^−1^
LPE(18:2)	−0.45	2.1 × 10^−4^	5.5 × 10^−3^	0.41	2.6 × 10^−2^	5.3 × 10^−1^
LPS(18:1)	−0.45	2.3 × 10^−4^	5.5 × 10^−3^			
LPS(18:0)	−0.44	2.7 × 10^−4^	5.6 × 10^−3^			
LPC(16:0)	−0.42	5.4 × 10^−4^	9.3 × 10^−3^	0.13	5.0 × 10^−1^	7.7 × 10^−1^
LPE(18:0)	−0.42	5.9 × 10^−4^	9.3 × 10^−3^	0.08	7.0 × 10^−1^	8.2 × 10^−1^
LPE(16:0)	−0.42	6.5 × 10^−4^	9.3 × 10^−3^	−0.13	4.9 × 10^−1^	7.7 × 10^−1^
TG(52:0)	0.41	8.5 × 10^−4^	1.0 × 10^−2^	−0.21	2.8 × 10^−1^	6.9 × 10^−1^
TG(54:0)	0.41	8.9 × 10^−4^	1.0 × 10^−2^			
TG(50:0)	0.41	9.4 × 10^−4^	1.0 × 10^−2^	−0.17	3.7 × 10^−1^	7.7 × 10^−1^
LPC(18:0)	−0.40	1.2 × 10^−3^	1.2 × 10^−2^	0.14	4.7 × 10^−1^	7.7 × 10^−1^
TG(48:0)	0.39	1.6 × 10^−3^	1.5 × 10^−2^	−0.12	5.3 × 10^−1^	7.7 × 10^−1^
LPE(20:4)	−0.37	2.9 × 10^−3^	2.6 × 10^−2^	0.22	2.5 × 10^−1^	6.9 × 10^−1^
SHexCer(d18:1/22:1)	0.36	3.8 × 10^−3^	3.1 × 10^−2^	−0.2	3.0 × 10^−1^	7.1 × 10^−1^
HexCer(d18:1/22:1)	0.36	3.8 × 10^−2^	3.1 × 10^−2^	−0.22	2.4 × 10^−1^	6.9 × 10^−1^
Hex2Cer(d18:1/16:0)	0.34	5.8 × 10^−3^	4.4 × 10^−2^	−0.15	4.5 × 10^−1^	7.7 × 10^−1^

*Note:* Lipid species that were significantly correlated between a common subset of matched serum and CSF samples (*n* = 60) in the discovery cohort (*q* < 0.05), and the corresponding results for the validation cohort (*n* = 29). Four lipids were not measured in the validation cohort. *r*: Pearson's correlation coefficient.

### 3.6 Association of Lipids With Clinical Indicators of Disease Activity

#### 3.6.1 Relapse Status

**FIGURE 4:**
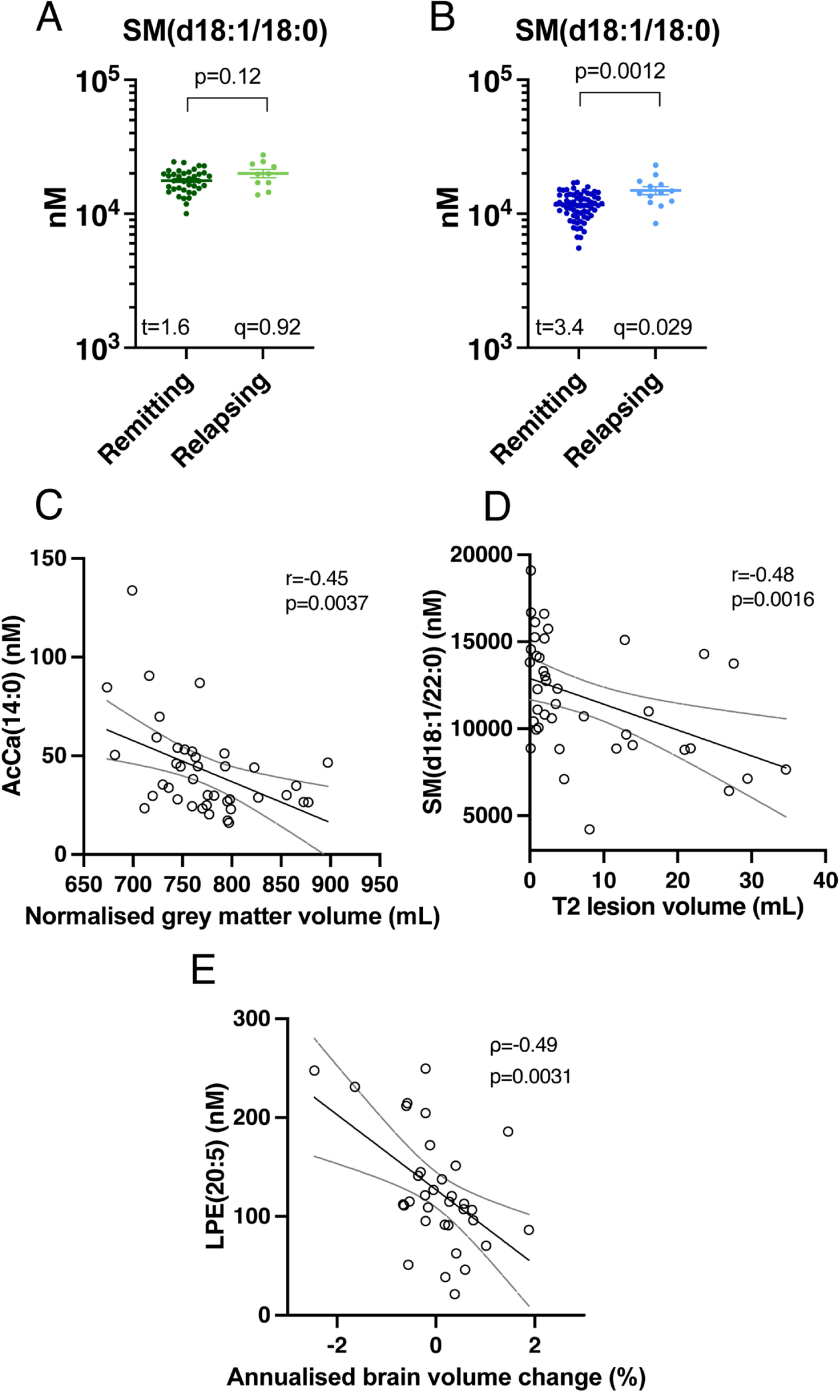
Lipids associated with relapse status or MRI metrics in the validation cohort. (A, B) Levels of SM(d18:1/18:0) in samples collected during a relapse compared to those in remission, in the discovery (A) and validation (B) cohort. Significance was assessed by *t*‐tests (A, B) adjusted for multiple comparisons. (C) Correlation of AcCa(14:0) with grey matter volume. (D) Correlation of SM(d18:1/22:0) with T2 lesion volume. (E) Correlation of LPE(20:5) with annualised brain volume change (%). Lines of best fit are shown, with 95% confidence intervals. r, Pearson's correlation coefficient. *ρ*, Spearman's correlation coefficient.

No lipids were significantly affected by relapse status in the discovery cohort at *q* < 0.05, whereas Hex2Cer(d18:1/16:0) (*t* = 3.3, *p* = 1.3 × 10^−3^, *q* = 2.9 × 10^−2^), SHexCer(d18:1/22:1) (*t* = 3.5, *p* = 8.6 × 10^−4^, *q* = 2.7 × 10^−2^), SM(d18:2/18:0) (*t* = 3.9, *p* = 1.8 × 10^−4^, *q* = 1.4 × 10^−2^), SM(d18:2/20:0) (*t* = 3.8, *p* = 2.8 × 10^−4^, *q* = 1.5 × 10^−2^), and SM(d18:1/18:0) (*t* = 3.4, *p* = 1.2 × 10^−3^, *q* = 2.9 × 10^−2^) were significantly higher; and LPE(20:5) (*t* = −4.4, *p* = 3.3 × 10^−5^, *q* = 5.1 × 10^−3^), LPC(20:5) (*t* = −3.6, *p* = 6.4 × 10^−4^, *q* = 2.5 × 10^−2^), and PC(36:5) (*t* = −3.2, *p* = 2.3 × 10^−3^, *q* = 4.5 × 10^−2^) were lower, when comparing the 13 RRMS cases in relapse to 68 in remission in the validation cohort. Of these, only SM(d18:1/18:0) showed a similar trend in the discovery cohort, although not statistically significant (Figure 4A,B). Combining the two cohorts, mean SM(d18:1/18:0) levels were significantly higher in samples taken during relapse, after adjusting for age, sex, collection site and treatment (logistic regression, *Χ* ^2^ = 34, OR per SD = 1.2 (95% CI = 1.1–1.3), *p* = 3.1 × 10^−3^).

#### 3.6.2 EDSS and MSSS

No lipids were correlated with EDSS or MSSS scores at *q* < 0.05 in the discovery cohort. In the validation cohort, AcCa(14:0), AcCa(16:0), AcCa(16:1), Cer(d18:1/18:0), and the myelin‐enriched 2‐hydroxy sphingolipids HexCer(d18:1/18:0(2OH)) and HexCer(d18:1/24:1(2OH)) were positively correlated with EDSS scores, while Cer(d18:1/18:0), C1P(d18:1/18:0), HexCer(d18:1/22:0(2OH)) and HexCer(d18:1/24:1(2OH)) were correlated with MSSS scores, adjusting for collection site (Table 5). Considering the treated and untreated MS cases in the validation cohort distinctly, a similar set of lipid species including AcCa(14:0), AcCa(16:0), Cer(d18:1/18:0), and HexCer(d18:1/18:0(2OH)) were positively correlated with EDSS at *p* < 0.05 regardless of treatment; however these associations did not survive FDR correction due to smaller sample sizes.

**TABLE 5:** Lipids significantly correlated with EDSS and MSSS in the validation cohort.

Lipid	EDSS^a^					MSSS^b^				
	Discovery		Validation			Discovery		Validation		
	*r*	*p*	*r*	*p*	*q*	*r*	*p*	*r*	*p*	*q*
AcCa(14:0)	−0.28	9.9 × 10^−1^	**0.42**	**1.7 × 10** ^**−5**^	**2.1 × 10** ^**−3**^	−0.21	2.2 × 10^−1^	0.32	1.7 × 10^−3^	5.3 × 10^−2^
AcCa(16:0)	−0.25	1.3 × 10^−1^	**0.41**	**2.7 × 10** ^**−5**^	**2.1 × 10** ^**−3**^	−0.13	4.5 × 10^−1^	0.31	2.7 × 10^−3^	7.0 × 10^−2^
AcCa(16:1)	−0.29	8.3 × 10^−1^	**0.33**	**1.1 × 10** ^**−3**^	**4.1 × 10** ^**−2**^	−0.21	2.2 × 10^−1^	0.26	1.3 × 10^−2^	1.2 × 10^−1^
C1P(d18:1/18:0)	ND	ND	0.28	5.5 **×** 10^−3^	7.9 × 10^−2^	ND	ND	**0.37**	**2.6 × 10** ^**−4**^	**2.0 × 10** ^**−2**^
Cer(d18:1/18:0)	−0.18	2.9 × 10^−1^	**0.38**	**1.6 × 10** ^**−4**^	**8.4 × 10** ^**−3**^	−0.08	6.2 × 10^−1^	**0.35**	**7.1 × 10** ^**−4**^	**3.0 × 10** ^**−2**^
HexCer(d18:1/18:0(2OH))	−0.10	5.6 × 10^−1^	**0.31**	**1.9 × 10** ^**−3**^	**4.9 × 10** ^**−2**^	−0.13	4.6 × 10^−1^	0.28	7.1 × 10^−3^	8.9 × 10^−2^
HexCer(d18:1/22:0(2OH))	−0.19	2.5 × 10^−1^	0.27	7.6 **×** 10^−3^	8.8 × 10^−2^	−0.23	1.7 × 10^−1^	**0.35**	**7.6 × 10** ^**−4**^	**3.0 × 10** ^**−2**^
HexCer(d18:1/24:1(2OH))	−0.27	1.0 × 10^−1^	**0.32**	**1.3 × 10** ^**−3**^	**4.1 × 10** ^**−2**^	−0.39	1.8 × 10^−2^	**0.43**	**2.1 × 10** ^**−5**^	**3.3 × 10** ^**−3**^

*Note: r*: Pearson's correlation coefficient; ND: not determined (the lipid was not measured in that cohort). Discovery cohort correlations were adjusted for age, while validation cohort correlations were adjusted for collection site. Bold indicates statistical significance at *q* < 0.05.

^a^ EDSS scores were available for 38/47 MS cases in the discovery cohort and 97/99 MS cases in the validation cohort.

^b^ MSSS scores were available for 38/47 MS cases in the discovery cohort and 92/99 MS cases in the validation cohort.

#### 3.6.3 MRI Parameters

Measures of T2 lesion number, lesion volume, whole brain volume, thalamic volume, and grey matter volume were obtained for 40 MS cases in the validation cohort. For 35 cases, prior scans allowed a determination of annualised percentage brain volume change; however one was determined to be an outlier and excluded. Although many lipids were associated with these MRI metrics at *p* < 0.05 (Figure S1), none of these remained significant at *q* < 0.05. Applying a less conservative cut‐off of *p* < 0.005, AcCa(14:0) was inversely associated with grey matter volume (*r* = −0.45) (Figure 4C), SM(d18:1/22:0) was inversely correlated with lesion volume (*r* = −0.48) (Figure 4D), and LPE(20:5) was inversely correlated with annualised change in brain volume (*ρ* = −0.49) (Figure 4E).

## 4 Discussion

In this study, we identified 25 lipids that were reproducibly higher in the serum of RRMS compared to HC subjects across two sample cohorts. All of these lipids were also higher in the OND group of the discovery cohort, likely reflecting the effect of neuroinflammatory diseases on the peripheral lipidome. This was particularly notable for SM(d18:1/18:0), which was not only higher in RRMS compared to HC, but also higher in RRMS samples taken during relapse and particularly high in the aggressive neuroinflammatory diseases NMOSD and ADEM. A combination of five lipids differentiated RRMS from HC with > 80% sensitivity in random forest models, but showed lower specificity and could not differentiate RRMS from OND or PMS. Eight lipids, including three AcCa's, three hydroxylated HexCers, Cer(d18:1/18:0) and C1P(d18:1/18:0), were correlated with EDSS and/or MSSS scores in the validation cohort; however these associations were not seen in the discovery cohort. Given their ease of measurement, future studies should determine if serum LPC, LPE, SM, and/or AcCa levels are increased in clinically isolated syndrome, the initial demyelinating episode that often precedes an MS diagnosis, and whether these lipids are useful biomarkers for relapses and disability progression.

Our observation of reproducibly increased Cer(d18:1/16:0) and HexCer(d18:1/16:0) levels in MS compared to control serum is consistent with previous findings in serum (Filippatou et al. 2021), plasma (Kurz et al. 2018), and CSF (Checa et al. 2015; Vidaurre et al. 2014). Since the C16 N‐acyl chain is not abundant in myelin sphingolipids (Teo et al. 2023) it is likely that increased serum levels of d18:1/16:0 Cer and HexCer are derived from peripheral sources such as the liver, adipose tissue and blood cells. Nonetheless, recent work has identified neuronal Cer(d18:1/16:0) as a driver of mitochondrial dysfunction, neurodegeneration and disability in experimental autoimmune encephalitis, a mouse model of autoimmune demyelination (Amatruda et al. 2025). Cer(d18:1/16:0) inhibits complexes of the electron transport chain, suppressing respiratory metabolism and increasing oxidative stress (Diaz‐Vegas et al. 2023), although another study implicated Cer(d18:1/24:0) as a key mediator of these processes in progressive MS (Wentling et al. 2019). Ceramides also initiate nuclear factor‐κB‐mediated transcription of genes encoding proinflammatory cytokines (Lee et al. 2023), mediated in part through the formation of plasma membrane microdomains that cluster and activate tumour necrosis factor superfamily and T‐cell receptors (Legler et al. 2003; Zhu et al. 2011).

SM(d18:1/18:0) and SM(d18:2/18:0), both of which were increased in RRMS compared to HC, are highly enriched in neuronal cell membranes (Ginkel et al. 2012). Increased serum SM(d18:1/18:0) during relapse may be indicative of neurodegeneration, and this should be investigated in future studies by correlating levels of this lipid with NfL. In an Alzheimer's Disease cohort, CSF SM(d18:1/18:0) was positively correlated with both NfL and the Alzheimer's‐specific biomarker phospho‐tau(181) (Morrow et al. 2022). Cer(d18:1/18:0), the metabolic precursor to SM(d18:1/18:0), was correlated with EDSS scores in our validation cohort, and is increased in human MS lesions and following cuprizone‐induced demyelination in mice (Kim et al. 2012).

Our finding of reproducibly increased levels of multiple LPC species in RRMS concurs with prior studies (Lattau et al. 2024; Momchilova et al. 2022; Villoslada et al. 2017), although others have reported reduced levels (Del Boccio et al. 2011; Ferreira et al. 2021; Stoessel et al. 2018). LPC promotes macrophage (Ousman and David 2000) and T‐cell recruitment (Radu et al. 2004), as well as inflammatory cytokine (Huang et al. 1999) and reactive oxygen species generation (Ojala et al. 2007). Lysophospholipids are also an indicator of recent demyelination (Penkert et al. 2021) and LPC itself induces localised demyelination. However, LPC levels were not increased in CSF of people with MS (Shi et al. 2024), suggesting that it is not directly associated with demyelination. In fact, omega‐3 fatty acid‐containing LPC(22:6) is required for myelin synthesis and must be actively transported into the CNS (Sengottuvel et al. 2023). Serum LPC(20:5) and LPE(20:5) levels were previously associated with EDSS progression from < 3 to > 4.5 after 2 years (Villoslada et al. 2017) but were not associated with EDSS or MSSS in our study. LPE(20:5) was correlated with brain atrophy in our validation cohort, although not significant at *q* < 0.05.

LPC and LPE can be generated via *de novo* lipid synthesis, phospholipase A_1_ and A_2_ activity, lecithin‐cholesterol acyltransferase, or endothelial and hepatic lipases that cleave lipoprotein‐associated PC (Law et al. 2019). In agreement with prior studies, PLA_2_ activity was higher in PMS compared to HC and RRMS (Sternberg et al. 2012), but not in RRMS compared to HC cases (Momchilova et al. 2022; Siroos et al. 2013), indicating that higher lysophospholipids in RRMS cannot be explained by the PLA_2_ activity that we measured. Statistically significant decreases in the glycerophospholipids PC, PE, PEp and PS were observed for RRMS cases in the discovery cohort, whereas mean levels of these lipids decreased non‐significantly in our validation cohort. Prior studies have also reported lower PC, PE, and PEp in RRMS serum samples (Ferreira et al. 2021; Villoslada et al. 2017), and PEp has been associated with lower relapse rates (Virupakshaiah et al. 2023). We note, however, that PEp levels were significantly lower in our OND group compared to both RRMS and HC, indicating that a reduction in serum PEp levels is not unique to MS.

No prior studies have reported significant changes to circulating or tissue AcCa levels in MS, although PLS‐DA models using AcCa's and amino acids in dried plasma spots differentiated MS from control cases with 81% sensitivity and 100% specificity (AUC 0.98) (Kasakin et al. 2020). AcCa(16:0) and AcCa(18:1) were significantly higher in RRMS in both of our cohorts, and three AcCa's, including AcCa(16:0), were correlated with EDSS and MSSS scores in the validation but not the discovery cohort. Several AcCa's, including AcCa(16:0), were inversely correlated with normalised grey matter volume; however these correlations did not survive FDR correction. Serum AcCa species were positively associated with relapse rates in a paediatric‐onset MS cohort (Virupakshaiah et al. 2023), supporting their potential as biomarkers of disease activity.

AcCa's are short‐lived intermediates formed to transport fatty acids across the mitochondrial membranes for β‐oxidation. Accumulation of particular AcCa's is used to diagnose inherited β‐oxidation deficiencies; however elevated AcCa levels could also indicate increased β‐oxidation of fatty acids to meet bioenergetic requirements (Jiang et al. 2024; Makrecka‐Kuka et al. 2017). Demyelination increases the amount of ATP needed to maintain neuronal resting potential, and higher serum AcCa levels might therefore reflect the utilisation of peripheral fat or myelin lipids as an energy source for the CNS and/or immune system. This would concur with higher serum LPC, LPE, and DG levels, which can result from fat lipolysis. Our observation of higher DG levels, including DG(16:0_18:1) and DG(18:0_18:1), in people with MS concurs with published studies using plasma and serum (Lattau et al. 2024; Villoslada et al. 2017), although another study reported no change to DG(18:1_20:4) (Meier et al. 2024).

Strengths of this study include the testing of all associations except correlations of lipids with MRI metrics in two independent RRMS sample cohorts, and the use of stringent FDR‐adjusted *p* values to account for multiple comparisons. Limitations include the absence of OND samples and a small PMS sample size in the validation cohort, and the absence of PMS cases in the discovery cohort. In addition, the lack of BMI for all samples prevented us from adjusting our analyses for the effect of BMI on lipid levels. EDSS scores were not available for 9/48 samples in the discovery cohort, which adversely affected statistical power, and MRI metrics were only available for 40 MS cases in our validation cohort, resulting in the absence of significant correlations after FDR correction. Another important limitation is the broad range of sample storage time for our discovery cohort samples (4–19 years at −80°C), which could affect lipid levels and PLA_2_ activity. In contrast most validation cohort samples had been stored for less than 1 year prior to analysis. Finally, we did not quantify levels of free fatty acids, which might clarify if higher lysophospholipids and AcCa's in RRMS are a lipolysis signature.

DG, Cer, LPC, and LPE levels were higher in the discovery compared to the validation cohort, whereas other lipid classes were consistent between the cohorts. This is likely to be at least partially attributed to differences in diet and lifestyle, as levels of these lipids are affected by obesity and diet. BMI was much higher in the discovery cohort and the serum samples were not from fasted individuals. BMI is associated with levels of many circulating lipids (Beyene et al. 2023; Xu et al. 2024) and has been associated with higher plasma ceramide levels in people with MS (Castro et al. 2019). However, while DG and Cer are positively correlated with BMI, lysophospholipids are inversely associated (Beyene et al. 2023; Xu et al. 2024), suggesting that BMI alone cannot explain the changes to serum lipids in people with RRMS. Cohort studies controlling for BMI and diet are needed to isolate the effects of these demographic variables from those of MS on serum lipids. To minimize variance in quantification between the cohorts, we used an internal standard for each lipid class and analyzed samples on the same instrument. However, we cannot rule out the possibility that differences between the cohorts result partly from sample collection, storage, and extraction or ionisation efficiency. Despite the differences in estimates of absolute abundance for certain lipids, we identified reproducible and highly significant differences in relative abundance of lipids between RRMS cases and controls.

## 5 Conclusions

This study has identified 25 Cer, SM, LPC, LPE, AcCa and DG species that were reproducibly increased in RRMS compared to HC. We speculate that increased levels of LPCs, LPEs, DGs, and AcCa's in RRMS might be indicative of increased lipolysis and consumption of fatty acids by β‐oxidation, perhaps in response to increased energetic demands or altered metabolic conditions. Increased SM(d18:1/18:0) may be indicative of severe neuroinflammation and/or neurodegeneration. Future studies should follow lipid levels over time in larger cohorts to clarify if any of these lipids are consistently associated with changes to disability scores, serum NfL, and lesional or grey matter volume.

## Author Contributions

**Lisa Shi:** conceptualization, data curation, formal analysis, investigation, writing – original draft, writing – review and editing, methodology. **Laura Ghezzi:** conceptualization, data curation, formal analysis, investigation, writing – review and editing, resources. **Georgia Watt:** formal analysis, investigation, writing – review and editing. **Drishya Mainali:** data curation, resources. **Dana Perantie:** data curation, resources. **Chiara Fenoglio:** data curation, resources. **Collin Tran:** data curation, resources. **Alexander Dupuy:** data curation, resources. **Freda Passam:** data curation, resources. **Monokesh K. Sen:** data curation, resources. **Humphrey Chan:** data curation. **Samuel Kwok:** data curation. **Chenyu Wang:** formal analysis, investigation, methodology. **Michael Barnett:** formal analysis, investigation, methodology. **Todd Hardy:** writing – review and editing, funding acquisition. **Laura Piccio:** conceptualization, data curation, formal analysis, investigation, supervision, funding acquisition, writing – review and editing. **Anthony S. Don:** methodology, supervision, writing – original draft, writing – review and editing, funding acquisition, investigation, formal analysis, conceptualization.

## Conflicts of Interest

M.B. is a consulting neurologist and honorary Research Director at Sydney Neuroimaging Analysis Centre (SNAC), the entity that produced iQ‐Solutions image analysis software. C.W. is a part‐time employee and holds equity ownership in SNAC. All other authors declare no conflicts of interest.

## Peer Review

The peer review history for this article is available at https://www.webofscience.com/api/gateway/wos/peer‐review/10.1111/jnc.70285.

## Supporting information


**Data S1:** Supporting Information.

**Data S2:** Supporting Information.

## Data Availability

The data that supports the findings of this study are available in the Supporting Information of this article.
