# Supplementary material for: The Serum Lipid Profile of Relapsing Multiple Sclerosis Differs Reproducibly From Healthy Controls
Source: J Neurochem. 2025 Nov 5;169(11):e70285. doi: 10.1111/jnc.70285 (PMC12589855; doi:10.1111/jnc.70285)
Supplement: Supplementary file 1 — Data S1: Supporting Information. [file JNC-169-0-s002.pdf]

The serum lipid profile of relapsing multiple sclerosis differs reproducibly from healthy controls

[1,2,3]Lisa Shi, [4,5]Laura Ghezzi, [1,2,3]Georgia Watt, [1,2,3]Drishya Mainali, [6]Dana Perantie, [4,5]Chiara Fenoglio, [3,7]Collin Tran, [1,8]Alexander Dupuy, [1,8,9]Freda Passam, [1,2,3]Monokesh K. Sen, [11]Humphrey Chan, [2,12]Samuel Kwok, [13]Chenyu Wang, [2,7,13]Michael Barnett, [10]Todd Hardy, [1,2,3]Laura Piccio, [1,2,3]Anthony S. Don.

[1]Charles Perkins Centre, The University of Sydney, NSW, Australia

[2]Brain and Mind Centre, The University of Sydney, NSW, Australia

[3]School of Medical Sciences, Faculty of Medicine and Health, The University of Sydney, NSW, Australia

[4]Department of Biomedical, Surgical and Dental Sciences, University of Milan, Milan, Italy

[5]Fondazione IRCCS Ca' Granda Ospedale Maggiore Policlinico, Milano, Italy

[6]Department of Neurology, Washington University, School of Medicine, St Louis, Missouri, USA

[7]Royal Prince Alfred Hospital, NSW, Australia

[8]Central Clinical School, Faculty of Medicine and Health, The University of Sydney, NSW, Australia

[9]Department of Haematology, Royal Prince Alfred Hospital, NSW, Australia

[10]Department of Neurology, Concord Hospital, NSW, Australia

[11]Illawarra Shoalhaven Local Health District, NSW, Australia

[12]Napean-Blue Mountain Local Health District, NSW, Australia

[13]Sydney Neuroimaging Analysis Centre, NSW, Australia

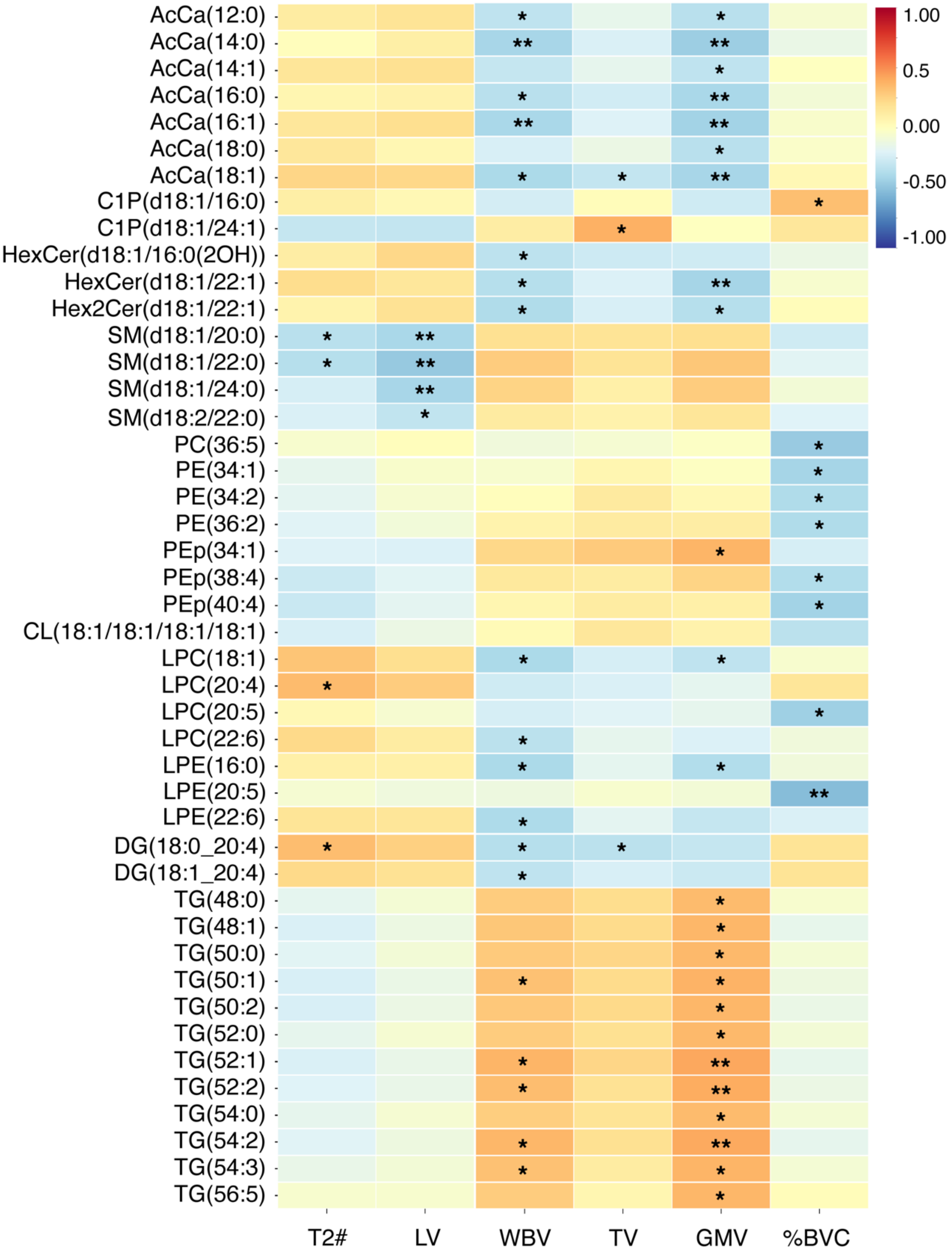

Supplementary Figure 1. Correlation of serum lipids with MRI volumetrics. Serum lipids that were significantly correlated with T2 lesion number (T2#), lesion volume (LV), normalised whole brain volume (WBV), normalised thalamic volume (TV), normalised grey matter volume (GMV) and percentage annualised brain volume change (%BVC) at univariate $p<0.05$ are shown; *$p<0.05$, **$p<0.01$. No asterisk indicates $p>0.05$. The colour scale shows the correlation coefficients (r, ρ). Correlations of lipids to T2#, LV, WBV and GMV were analysed by Pearson's correlation, while correlations to TV and %BVC were by Spearman's rank correlation. CL, cardiolipin.
